# Pernicious anemia presenting as pancytopenia

**DOI:** 10.1002/jha2.281

**Published:** 2021-10-20

**Authors:** Nwabundo Anusim, Vonda Douglas‐Nikitin, Ishmael Jaiyesimi

**Affiliations:** ^1^ William Beaumont Hospital, Hematology and Oncology; ^2^ William Beaumont Hospital, Pathology

## 1 PERNICIOUS ANEMIA PRESENTING AS PANCYTOPENIA

**FIGURE 1:**
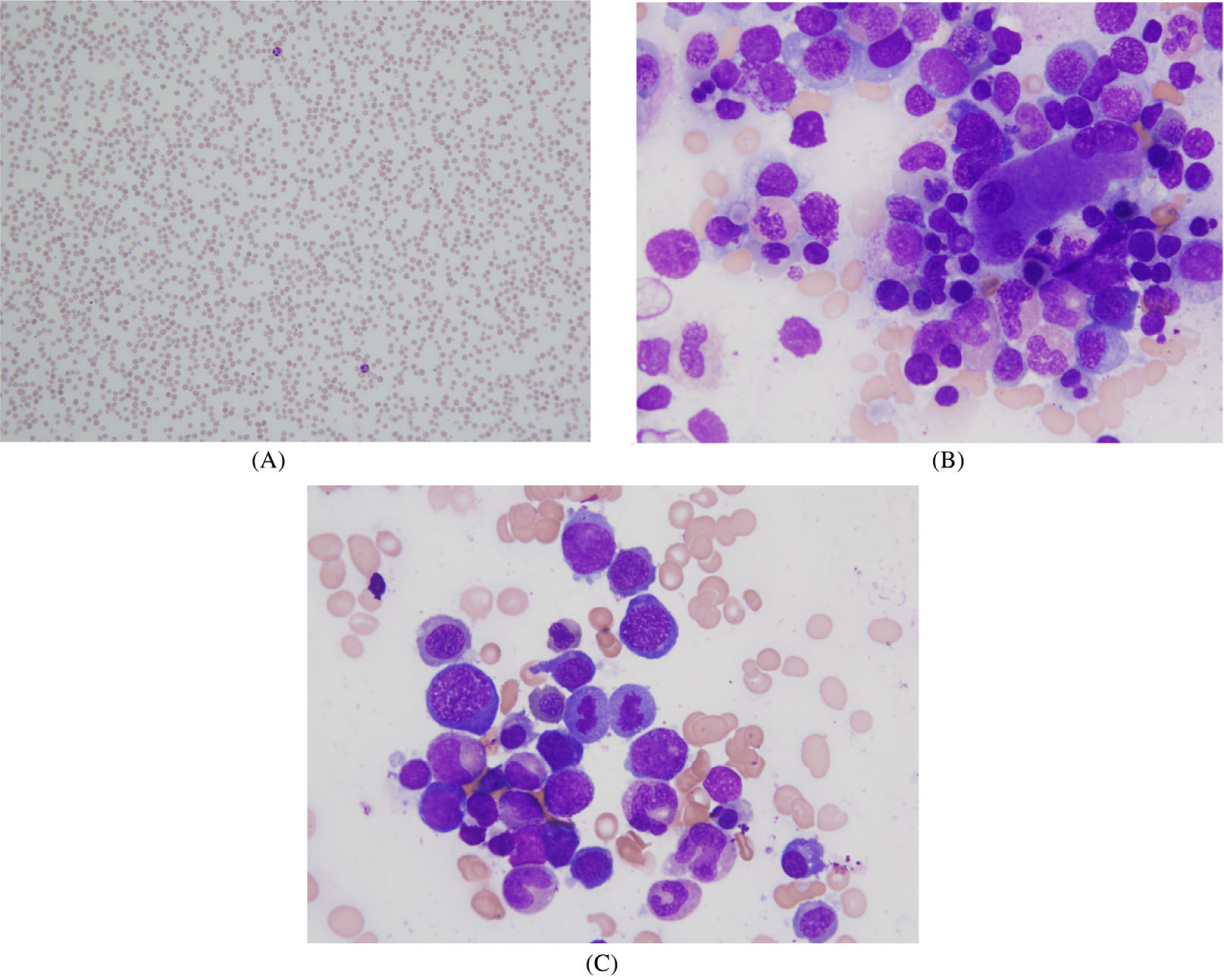
(A) Aspirate: Megaloblastic changes and atypical megakaryocytes. (B) Peripheral blood: pancytopenia. (C) Trilineage dysplasia

A 33‐year‐old male with no medical conditions presented to the emergency room with complaints of shortness of breath both on exertion and at rest. His symptoms had progressively worsened for the past 3 months with associated right‐sided headaches relieved with NSAIDS, epigastric pain and an episode of black stool. He drinks alcohol socially and denied use of nitrous oxide. His vital signs were stable, and examination was benign with no rash, hepatosplenomegaly or lymphadenopathy. Investigations revealed: WBC 2.7 × 10^9^ (reference range 3.5–10 × 10^9^/L), absolute neutrophil count 1100 (1500–8000/mm^3^). haemoglobin 3.6 (8.35–10.55 mmol/L), platelets 141 × 10^9^/L (150–400 × 10^9^/L), MCV 9.5 x 10^‐14^ (8.0–10.0 × 10^‐14^ L/cell) iron 13.25 (8.05–28.64 μmol/L), ferritin 1914 (31.5–759.5 pmol/L), haptoglobin < 0.08 (0.40–1.20 g/L), folate 42.5 (>11.3 nmol/L), vitamin B12 101.1 (200–642 pmol/L) and LDH > 70 (2.33–4.67 ukat/L). A peripheral blood smear showed pancytopenia, and bone marrow aspirate and biopsy revealed megaloblastic changes, atypical megakaryocytes and trilineage dysplasia respectively (Fig 1a,b,c). He had a methylmalonic acid level of 39 (≤0.40 μmol/mmol creatinine) and positive antibodies to intrinsic factor and parietal cells. A diagnosis of pernicious anaemia was made, and he received a week of vitamin B12 injections followed by high dose oral vitamin B12 daily. Due to his black stools and the high incidence of gastric malignancies associated with pernicious anaemia, he underwent an esophagogastroduodenoscopy which showed chronic atrophic gastritis. Laboratory results repeated after two weeks were normal.

## CONFLICT OF INTEREST

The authors declare that there is no conflict of interest that could be perceived as prejudicing the impartiality of the research reported.

